# Bariatric surgery improves knee function and not knee pain in the early postoperative period

**DOI:** 10.1186/s13018-018-0803-4

**Published:** 2018-04-11

**Authors:** Amre Hamdi, Alia T. Albaghdadi, Bayan Ghalimah, Abdullah Alnowiser, Anas Ahmad, Abdulmalik Altaf

**Affiliations:** 1 Department of Orthopedic Surgery, Faculty of Medicine, King Abdulaziz University, P.O Box 80215, 21589 Jeddah, Kingdom of Saudi Arabia; 2 Faculty of Medicine, King Abdulaziz University, Jeddah, Kingdom of Saudi Arabia; 3 Department of General Surgery, Faculty of Medicine, King Abdulaziz University, Jeddah, Kingdom of Saudi Arabia

**Keywords:** Knee pain, Arthritis, Knee function, Bariatric surgery, Rapid weight loss

## Abstract

**Background:**

Obesity remains the strongest predictor of knee osteoarthritis (OA). Studies have reported improvement in knee pain and function post-bariatric surgery secondary to weight loss and reduced mechanical loading, yet others found increased rates of total knee arthroplasty (TKA) in that patient population. To address this controversy, our study aimed to further assess the effect of surgically induced, “rapid” weight loss on knee pain and function.

**Methods:**

Obese patients with chronic knee pain, who were undergoing bariatric surgery, were enrolled and surveyed preoperatively and 3 months postoperatively. Our outcome measures were knee pain and knee function, assessed by a knee injury and osteoarthritis outcome score (KOOS). The paired *t* test was used to compare pre- and postoperative KOOS scores. Pearson correlation coefficient was used to test the correlation between change in body mass index (BMI) with knee function, pain, and stiffness.

**Results:**

A total of 30 patients was included in the study. The mean age was 35 years, with a mean preoperative BMI of 42.8. The mean difference in BMI at 3 months was 8.4 (SD3). There was a significant improvement in KOOS, − 23.2 (± 20) points, *p* < 0.01, most pronounced in knee function related to sport activities, with a difference of − 22.6 points, *p* < 0.01. Knee pain scores improved but did not reach statistical significance.

**Conclusion:**

Surgically induced rapid weight loss significantly improved knee function, particularly related to sports. However, there was no change in knee pain. This may be related to increased high-impact knee exercises and reduced lean mass. Tailored exercise programs for bariatric surgery patients postoperatively, may improve symptoms and decrease the need for knee replacements in the long term.

## Background

Knee osteoarthritis (OA) is one of the leading causes of knee pain and disability. A substantial escalation in the prevalence of knee pain and symptomatic knee OA was witnessed in the past two decades. The rates were found to double in women and triple in men [1]. The overall prevalence of knee pain in the US adult population is 20%, with over 61 million people affected [2], and a projected increase in prevalence to 25% by the year 2030 [3]. National data on the prevalence of knee pain and knee OA in Saudi Arabia is limited; however, significant numbers were reported ranging from 13 to 60% of studied sample populations in some regions of the country [4, 5], despite being a country with a relatively young population. Older age, female gender, smoking, diabetes, knee injury, and physical workload [6] such as squatting, kneeling, and lifting are among many known risk factors that contribute to the development and progression of knee OA. Nevertheless, obesity (BMI > 30 kg/m^2^) remains the strongest predictor of OA [7], while it is a modifiable risk. The mechanism by which obesity leads to knee joint damage and hence symptomatic OA is a complex process that includes biomechanical factors and an inflammatory process. Increased joint loading on mechanoreceptors overlying articular cartilage trigger a cascade of events resulting in cartilage degeneration. In addition, metabolic derangements in obese individuals lead to a systemic inflammatory response and hence disordered cartilage homeostasis [8]. Loss of hyaline cartilage is often accompanied with bony remodeling and peri-articular muscle weakness. This results in synovitis, periosteal irritation, and therefore knee pain [9].

With lifestyle modification, every 1 kg in weight reduction was found to be associated with more than twice the reduction in peak knee load [10]. Further, a 2-unit reduction in BMI over a 10-year period was associated with decreased odds for developing symptomatic knee OA by over 50% [11]. Amidst the alarming rates of the obesity epidemic that affects over 28% of the Saudi population [12], more people are resorting to a rather more “rapid” method of weight loss—bariatric surgery. Bariatric surgery proved to be a success, not only in weight reduction, but also in managing co-morbid conditions associated with obesity, such as diabetes, hypertension, and obstructive sleep apnea [13]. As one might expect, significant improvement in knee pain and function following bariatric surgery was also reported in some studies despite using different measurement instruments [14–16]. This was attributed to decreased mechanical loading and reduced markers of inflammation. Contrary to these findings, Trofa et al. [17] reported that bariatric surgery patients who are known to have arthritis are at an increased risk of requiring a total knee arthroplasty (TKA) following rapid weight loss. They suggested an active lifestyle on an already damaged knee as a possible culprit. In addition, the literature advocates for bariatric surgery as a staging tool prior to other non-bariatric surgical procedures in obese individuals [18], thereby lowering the risk and improving outcomes of a second indispensable surgery such as total knee replacement (TKR) [19].

To address the current controversy, we aimed at assessing the impact of rapid weight loss on the progression of knee pain and the development of OA in patients who undergo a bariatric procedure. We hypothesize that patients with a surgically induced rapid weight loss engage in more knee-loading activities, resulting in an “apparent” improvement in knee function but not knee pain. Further, we chose to select patients who suffer from knee pain, but without an identifiable cause or any radiographic evidence of OA, that is, who are not candidates for TKR, to limit the risk of bias in temporality.

The objectives of this study were to:

- Assess the change in knee pain and function pre- and postoperatively in patients undergoing bariatric surgery using the knee injury and osteoarthritis outcome score (KOOS) [20, 21].
- Determine the clinical significance of the change in the KOOS score in our patient population relative to a predetermined minimal clinically important change (MCIC) [22] of 8–10 points.

## Methods

We obtained ethical approval from the Institutional Review Board at King Abdulaziz University Hospital (KAUH), Jeddah, Saudi Arabia (Reference No. 322-14). We then took an informed consent from all patients prior to their enrollment into the study. We carried out the study in a single tertiary care center over a period of 6 months (July–December 2015). Patients were enrolled from the bariatric surgery clinic.

We used a quasi-experimental study design, where we surveyed our enrolled subjects preoperatively and 3 months postoperatively during their follow-up visit. Eligibility for enrollment were as follows: age > 18 years, candidate for a bariatric procedure as defined by the American Society for Metabolic and Bariatric Surgery (ASMBS) guideline [23] (BMI ≥ 40 or BMI ≥ 35 with at least two obesity-related co-morbidities), and the presence of self-reported chronic knee pain, without an identifiable pathology on clinical and radiographic evaluation. Patients who had a history of knee surgery and knee trauma and any evidence of OA and those on chronic use of nonsteroidal anti-inflammatory drugs (NSAIDS), or received intra-articular steroid injections, were excluded from the study.

Eligible patients underwent the usual pre-operative workup followed by either restrictive (sleeve gastrectomy) or a malabsorptive procedure (Roux-en-Y gastric bypass) depending on their initial weight, risks, and preference. A board-certified, experienced bariatric surgeon operated on all enrolled patients. Standardized pre- and postoperative protocol was used for all patients. No adverse events were noted. The mean length of hospital stay was 3 days. There were no intra-operative or postoperative complications.

Our primary outcomes were knee pain and knee function. These were assessed by the KOOS [20, 21], which is a validated, self-administered survey that takes about 15 min to complete. It is composed of five subscales: Knee Pain, Stiffness, Function in activity of daily living (ADL) [24], Function in sport and recreation (Sport/Rec), and knee-related Quality of life (QOL). Each question is made up of a 5-point Likert-type scale. An Arabic-validated translation of the survey was given to patients [25]. All patients were fluent Arabic speakers. The KOOS subscale “sport and recreation function” is more sensitive and discriminative than the Western Ontario and McMaster University Osteoarthritis Index (WOMAC) subscales, and therefore, we decided to use the KOOS instrument [20, 21]. Data from surveys were then administered into a publicly available online software [26] to calculate the total KOOS and the subscale scores.

Statistical analysis was conducted using STATA [27], version 13.1. Categorical variables were expressed as a number and percentage. Data variables were then tested for normality. Continuous variables were expressed as mean (SD) for normally distributed variables and as median (IQR) for skewed distributions. Paired *t* test was used to compare within group difference in pre- and postoperative mean scores. Pearson correlation co-efficient, *r*, was used to test correlations between independent variables. A two-tailed *p* value of < 0.05 was considered statistically significant.

## Results

### Patient demographics

A total of 30 patients met our selection criteria and were enrolled in our study, of which 24 were females (80%). Patient demographic characteristics are presented in Table 1. The mean age of patients was 35 years (SD 9), with a mean BMI of 42.7 (SD 5.73). Four patients were diabetic (13%), and 4 had a history of tobacco use (13%); 2 were current smokers, and 2 were ex-smokers. Patients had no history of trauma or knee surgery prior to the bariatric surgery. The majority of patients underwent sleeve gastrectomy (*N* 28, 9%), and 2 patients underwent gastric bypass surgery. No other bariatric procedures were done.

**Table 1:** Patient demographic characteristics

Patients demographics	*N*, % (total 30)
Age (mean, SD)	35.6 (9.3)
Gender	
Males	6 (20.0)
Females	24 (80.0)
Pre-op BMI (mean, SD)	42.8 (5.8)
Diabetes	4 (13.3)
History of smoking	4 (13.3)
Type of bariatric surgery	
Sleeve gastrectomy RYG bypass	28 (93.3)
	2 (6.7)

*BMI* body mass index, *Pre-op* preoperative, *RYG* roux-en-Y

### Knee function and knee pain

**Fig. 1:**
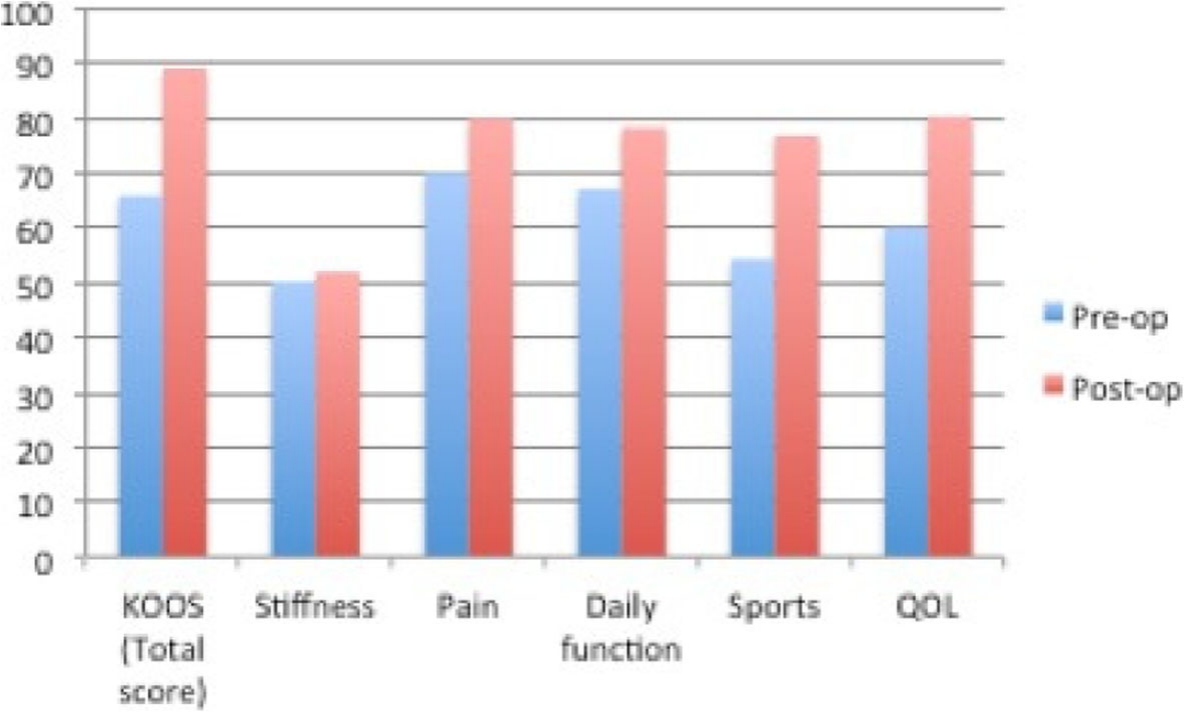
Comparison between mean KOOS sub-scores pre- and post-bariatric surgery

As shown in Table 2, there is a significant reduction in BMI 3 months postoperatively, with a mean difference of 8.4 (SD 3) units, *p* < 0.01, at 95% CI. Further, there is an overall significant reduction in the mean KOOS score, with a mean difference of − 23.2 (± 20) points, *p* < 0.01. The drop was most pronounced in knee function related to sport activities, with a difference of − 22.6 points, *p* < 0.01. Knee pain (*p* 0.147), stiffness (*p* 0.694), and daily function (*p* 0.167) improved, but not significantly from the preoperative period (Fig. 1). There were no difference in scores with regard to age, gender, diabetes, smoking status, or the type of bariatric surgery performed.

**Table 2:** Paired *t* test on BMI and KOOS pre- and post-op

	Pre-op (mean, SD)	Post-op (mean, SD)	*p* value (95% CI)
BMI	42.8 (5.7)	34.4 (5.3)	< 0.01
KOOS (total score)	66.0 (19.9)	89.3 (8.91)	< 0.01
Knee stiffness	50.2 (18.4)	52.0 (19.1)	0.694
Knee pain	70.3 (24.38)	79.9 (25.9)	0.147
Daily function	67.2 (26.51)	78.5 (28.9)	0.167
Sports function	54.3 (23.41)	77.0 (20.2)	< 0.01
QOL related to knee	60.2 (27.68)	80.3 (21.9)	0.004

*Pre-op* preoperative, *Post-op* postoperative, *BMI* body mass index, *KOOS* knee osteoarthritis outcomes score, *QOL* quality of life

Clinical significance of the improvement in the KOOS score was determined using a pre-defined value of the minimal clinically detectable change (MCIC of 10) [22] to dichotomize the change in KOOS score. Sixty-three percent of patients (*n* 19) had a clinically significant improvement in their KOOS scores.

### Correlation between change in BMI with knee function, pain, and stiffness

**Fig. 2:**
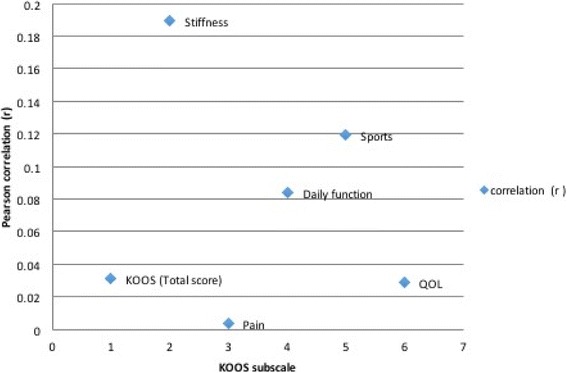
Pearson correlation co-efficient (*r*) of KOOS sub-scores with change in BMI

As shown in Table 3, the change in BMI was positively correlated with all KOOS parameters. Despite the lack of statistical significance, the highest correlation was seen in the improvement in knee stiffness and sport function with the change in weight. Contrary, the least parameter correlated with the change in weight was the change in knee pain (*r* 0.0039, *p* 0.983) (Fig. 2).

**Table 3:** Correlation between the change in BMI with the change in KOOS sub-scores

Change in KOOS score	Pearson correlation (*r*) with change in BMI	*p* value
KOOS (total score)	0.0314	0.869
Stiffness	0.1894	0.316
Pain	0.0039	0.9836
Daily function	0.0842	0.6581
Sports	0.1196	0.529
QOL	0.0289	0.8794

*BMI* body mass index, *KOOS* knee osteoarthritis outcomes score, *QOL* quality of life

## Discussion

Knee pain is an increasingly common complaint, particularly in obese individuals. Weight reduction lessens mechanical loading on knees. Bariatric surgery, an effective approach to obesity that results in rapid weight loss, is further expected to positively affect knee symptoms. Multiple studies reported a substantial improvement in knee pain and function using different measurement instruments following bariatric surgery [14–16]. Albeit prior findings, Trofa et al. [17] found an increased risk of TKR in arthritis patients 25–48 months following bariatric surgery. To address the current controversy, in this study, we aimed at further assessing the impact of rapid weight loss on knee pain and function.

Our results demonstrated a significant improvement in knee function related to sports, but insignificant change in knee pain, which is contrary to previous findings in the literature. Our results are supportive of the finding of Trofa et al. [17] in that our patients may later become candidates for TKR. The patients’ current more active lifestyle and increased demand on their knees since they are now more flexible due to weight loss as evident in our data may explain the insignificant change in their knee pain. The KOOS sport-related function subscale consists of five questions that assess the level of difficulty in squatting, running, jumping, twisting, and kneeling. All of which activities are known to cause patellofemoral pain syndrome (aka anterior knee pain) [28], which is a condition mainly described in active, young females [29]. Hence, the “rapid” transition from an inactive, sedentary life to a more active lifestyle may negatively impact long-term knee outcomes. Another possible explanation is that only a few patients maintain their lean body mass following bariatric surgery despite being active in exercise programs [30]. Moreover, rapid weight loss decreases dynamic and static muscle strength [31], and loss of quadriceps strength results in increased knee pain and the risk of arthritis development [31].

Given the importance of maintaining muscle mass and quadriceps strength with exercise, yet avoid activities that could induce even further damage with increased knee loading, a multidisciplinary care should be provided to patients postoperatively. There is a need to develop a standardized exercise protocol post-bariatric surgery to prevent further progression of knee pain and osteoarthritis and possibly avoid the need for a joint replacement.

Our study had several limitations; the lack of randomization limits causality; nevertheless, we attempted to control for confounders of knee pain by using a strict selection criteria. Patients were thoroughly evaluated prior to enrollment to exclude any identifiable knee pathology. In addition, the lack of significant improvement in knee pain may be secondary to our small sample size and short follow-up period. Although our follow-up period was limited to 3 months, reporting of data early in the postoperative period will allow us to examine sequential trends in the progression of our patients’ knee outcomes relative to bariatric surgery. Further, in this study, we used the KOOS that has a sport-related knee function subscale more sensitive and discriminative than the WOMAC subscales.

## Conclusion

In conclusion, the rapid transition to an active lifestyle seen with massive weight loss post-bariatric surgery may halt the expected improvement in knee pain. There is a need for tailored exercise programs for bariatric surgery patients to strengthen their muscles, preserve their lean mass, and yet prevent progression of knee pain from excess strain.

## Abbreviations

ADL: Activity of daily living
ASMBS: American Society for Metabolic and Bariatric Surgery
BMI: Body mass index
KAUH: King Abdulaziz University Hospital
KOOS: Knee Injury and Osteoarthritis Outcome Score
MCIC: Minimal clinically important change
NSAIDS: Nonsteroidal anti-inflammatory drugs
OA: Osteoarthritis
QOL: Quality of life
TKA: Total knee arthroplasty
TKR: Total knee replacement
WOMAC: Western Ontario and McMaster University Osteoarthritis Index
